# Using a Bayesian spatiotemporal model to identify the influencing factors and high-risk areas of hand, foot and mouth disease (HFMD) in Shenzhen

**DOI:** 10.1371/journal.pntd.0008085

**Published:** 2020-03-20

**Authors:** Xiaoyi He, Shengjie Dong, Liping Li, Xiaojian Liu, Yongsheng Wu, Zhen Zhang, Shujiang Mei

**Affiliations:** 1 Shantou University Medical College, Shantou, China; 2 Orthopedic Department, Yantaishan Hospital, Yantai, Shandong, China; 3 Shenzhen Center for Disease Control and Prevention, Shenzhen, China; Yale University, UNITED STATES

## Abstract

**Background:**

The epidemic of hand, foot, and mouth disease (HFMD) has become a severe public health problem in the world and has also brought a high economic and health burden. Furthermore, the prevalence of HFMD varies significantly among different locations. However, there have been few investigations of the effects of socioeconomic factors and air pollution factors on the incidence of HFMD.

**Methods:**

This study collected data on HFMD in Shenzhen, China, from 2012 to 2015. We selected eleven factors as potential risk factors for HFMD. A Bayesian spatiotemporal model was used to quantify the influence of the factors on HFMD and to identify the relative risks in different districts.

**Results:**

The risk factors of HFMD were the population, population density, concentration of SO_2_, and concentration of NO_2_. The relative risks (*RRs*) were 1.00473 (95% *CI*: 1.00059–1.00761), 1.00010 (95% *CI*: 1.00002–1.00016), 1.00215 (95% *CI*: 1.00170–1.00232) and 1.00058 (95% *CI*: 1.00028–1.00078), respectively. The protective factors against HFMD were the per capita GDP, the number of public kindergartens, the concentration of PM_10_, and the concentration of O_3_. The *RRs* were 0.98840 (95% *CI*: 0.98660–0.99026), 0.97686 (95% *CI*: 0.96946–0.98403), 0.99108 (95% *CI*: 0.98551–0.99840) and 0.99587 (95% *CI*: 0.99534–0.99610), respectively. The risk of incidence in Longgang district and Pingshan district decreased, while the risk of incidence in Baoan district increased.

**Conclusions:**

Studies have confirmed that socioeconomic factors and air pollution factors have an impact on the incidence of HFMD in Shenzhen, China. The results will be of great practical significance to local authorities, which is conducive to accurate prevention and can be used to formulate HFMD early warning systems.

## Author summary

This study used Bayesian spatiotemporal modelling to analyze the factors that influence the incidence of hand, foot, and mouth disease (HFMD)in Shenzhen, including socioeconomic factors and air pollution factors. A total of 171,210 HFMD cases and socioeconomic and air pollution data from 2012–2015 were included in the analysis. Studies have confirmed that socioeconomic factors and air pollution factors affect the risk of HFMD in Shenzhen, China. By testing the optimal model, the relative risk map of each area was drawn to identify the high-risk areas of HFMD and the future trends of the disease. The results will be of great practical significance to local authorities, which is conducive to accurate prevention and can be used to formulate targeted disease intervention measures.

## Introduction

Hand, foot and mouth disease (HFMD) is a common viral disease in infants and children under 5 years of age. This infection is caused by polio-enterovirus infection. Coxsackievirus A16 (Cox A16) and Enterovirus 71 (EV 71) are the most common etiologic agents [1,2]. The specific clinical manifestations of HFMD include oral pain, anorexia, and fever, and the hands, feet, mouth or other areas of the body will appear to have small sores or ulcers [3]. Usually, HFMD can heal itself, and some patients can heal without medication within about a week. Therefore, its clinical burden and health hazards are often neglected.

There have been millions of cases of HFMD around the world, and it formed a large-scale epidemic on a global scale [4–10]. The study also showed that the average cost (USD) of outpatients with mild infections, inpatients with mild infections, severe cases and fatal cases of HFMD were $201 (95% CI $187, $215), $1072 (95% CI $999, $1144), $3051 (95% CI $2905, $3197) and $2819 (95% CI $2068, $3571), respectively [11]. Therefore, HFMD epidemics has become a serious public health problem and has also brought a high economic and health burden.

The incidence of HFMD in all age groups in mainland China from 2009 to 2014 were 86.59/100000, 132.35/100000, 120.21/100000, 160.17/100000, 134.37/100000, and 203.16/100000, respectively [12]. According to the communicable disease surveillance system of Guangdong Province, China, the numbers of reported cases of HFMD from 2008 to 2013 were 48917, 93067, 226622, 274006, 330621 and 358068, respectively [13].

Although the first EV-A71 vaccine was already on the market in China, this vaccine could not protect against other novel emerging etiologies of HFMD. Hence, there is no general vaccine available for HFMD [14,15]. Therefore, identifying its influencing factors and working toward early warning and timely prevention are still the keys to reducing the severity of the incidence of HFMD [16].

Research over the past decade has repeatedly verified the association between HFMD epidemics and climatic factors [17]. However, due to rapid urbanization and climate change in recent years, severe and sustained air pollution has also led to an increase in morbidity and mortality [18–20]. In the past, research on the adverse health effects of air pollution has focused on the relationship between air pollution and chronic noncommunicable diseases [21,22]. However, recent studies have shown that air pollution may affect the incidence of infectious diseases [23–26]. The adhesion of the virus to gaseous particulate matter and the dissolution of substances such as NO_2_ in the respiratory tract may promote the occurrence of infectious diseases [22]. Enteroviruses attached to environmental particles can be transported longer distances under favorable weather conditions [27]. However, there have been few studies of the relationship between air pollution and HFMD thus far. Understanding the influence of air pollution factors, social factors and their combined effects on HFMD will help the relevant departments formulate more accurate and practical preventive measures.

In the past, most studies have used time-series models (SIR, ARIMA) to estimate the impacts of variables [28–30]. In recent years, since the discovery that logistic regression cannot explain spatial heterogeneity and spatial correlation well, it is recommended that the Bayesian spatiotemporal model be used to identify spatiotemporal variations and the effects of potential predictors [31–33]. The method uses Markov chain Monte Carlo simulation to estimate the posterior distribution of random variables and to identify the relative risks in different districts. As a result, the relevant departments can adequately implement preventive measures in high-risk areas, reduce the harm of HFMD and protect public health.

We aimed to quantify the influence of factors on HFMD and to discuss the possibility of improving the early predicted warning system in Shenzhen by using the Bayesian spatiotemporal model in this study.

## Methods

### Ethics statement

Not applicable. The Ethics Committee of Shantou University Medical College declared that this study did not require ethics approval.

### Data collection

#### Study area

**Fig 1:**
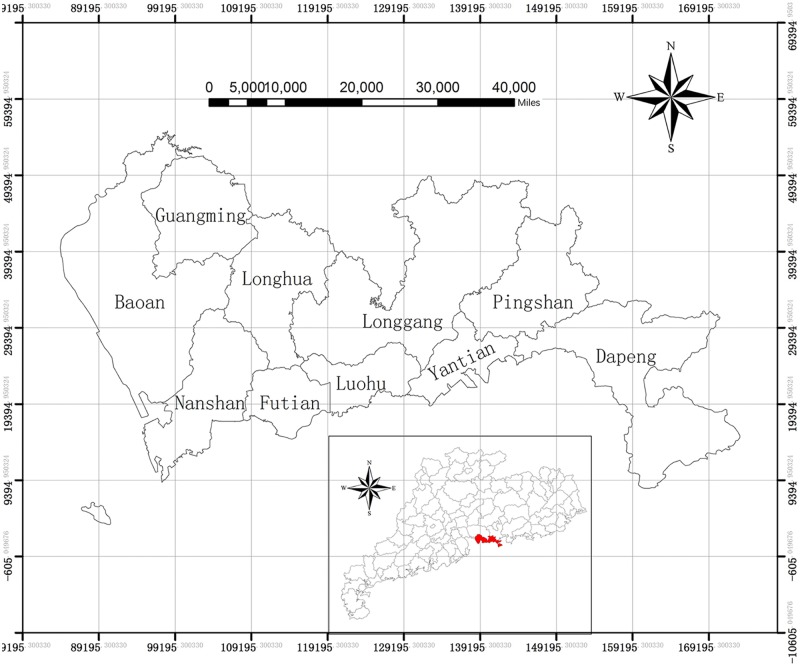
Shenzhen city and its location in Guangdong province. This figure was generated using ArcGIS Geographic Information Systems software version 10.2 (ESRI, USA).

Shenzhen (Fig 1) is located in the southern part of Guangdong Province and includes 10 administrative districts (Yantian, Luohu, Futian, Nanshan, Dapeng, Longhua, Longgang, Pingshan, Baoan, and Guangming). It is the central city in China’s economy, and its total economy ranks third in mainland China. Moreover, Shenzhen city, as an immigrant metropolis connecting China and Hong Kong, has a high population density and strong liquidity. These characteristics have also led to a high incidence of HFMD in Shenzhen over the years that is four times the national average [34].

Yantian, Luohu, Futian, and Nanshan are the economic core areas of Shenzhen and are the former Shenzhen special economic zones. Yantian, Luohu and Futian are also on the border with Hong Kong, which is a transport hub for Shenzhen and Hong Kong. The remaining six districts (Dapeng, Longhua, Longgang, Pingshan, Baoan, and Guangming) have been developed in the past 20 years and have large populations, but the economic level is lower than those of the other 4 districts (Yantian, Luohu, Futian, and Nanshan).

#### Surveillance data of HFMD

Case-based HFMD surveillance data from 2012 to 2015 were obtained from the Shenzhen Center for Disease Control and Prevention (Shenzhen CDC). The clinical criteria for the diagnosis of HFMD were provided by a guidebook published by the Ministry of Health of China in 2009 [35]. Since May 2, 2008, HFMD has been included in the class C infectious diseases. When patients are diagnosed with HFMD, all kinds of medical institutions are required to register the cases on the Notifiable Infectious Diseases Reporting Information System (NIDRIS) within 24 hours of diagnosis. The reports include the patient information, including name, age, gender, address, symptoms, and date of onset.

HFMD is a kind of infectious disease that legally must be reported. The Shenzhen Center for Disease Control and Prevention organize an investigation into underreporting by medical institutions every year [36]. In addition, the outpatient log and the hospital records of the medical institutions in Shenzhen basically implement electronic data collection [37]. It is also beneficial to the self-investigation of medical institutions and the integrity of the collection of HFMD information. Therefore, the data source of this study is accurate and reliable.

#### Potential predictor data

Previous studies have confirmed the relationship between demographic factors, socioeconomic factors, air pollution factors and the risk of HFMD [19,24,26,29,38]. Some studies have also established models to predict the incidence of HFMD through these factors [19,29,38,39]. However, at present, no model uses the above factors to predict the incidence of HFMD. The more comprehensive the influencing factors considered in the model, the more accurate the prediction effect will be.

The average concentration of SO_2_, the average concentration of NO_2_, the average concentration of PM_10_, and the average concentration of O_3_ were obtained from the Shenzhen environmental protection station, and no data were missing for the study period.

The “Shenzhen Yearbook” collects substantial social and economic information, including the population, geographical area, GDP (gross domestic product), and number of kindergartens. The population density is calculated by the population and area. The per capita GDP is calculated by the population and GDP.

The ESRI ArcGIS software (U.S. Environmental Systems Institute) was used to plot the spatial distribution of the data.

#### Incidence rate standardization

1. Because the populations of each district in Shenzhen were different from 2012 to 2015, it was necessary to standardize the annual incidence of each district. Finally, the average value was used as an index to evaluate the incidence of disease in each district for 4 years. The average incidence rate (*p*) in Shenzhen city from 2012 to 2015 was used as the standard;
2. The expected number of cases were calculated as follows: *p* *n_i,k_ (n_i,k_ represents the corresponding population, where index i represents the different districts, and index k represents the different years);
3. The standardized rate was calculated as follows: (P_i,k_ / *p* *n_i,k_)* (n_i,k_ /N_k_), where P_i,k_ represents the actual number of cases and N_k_ represents the population of Shenzhen.
4. The average value of each district was calculated over the 4 years.

#### Identification of risk variables

To assess the multicollinearity between the independent variables and select the variables that were included in the model, we used R software version 3.4.4 to calculate the variance inflation factor (VIF) between the variables.

#### Statistical analysis

The HFMD cases (*O*_*i*,*k*_) for the i-th district were assumed to have a Poisson distribution with mean *μ*_*i*,*k*_ · (*i* = 1,2,…,10; *k* = 1,2,…,4). Index i represents the different district (space), and index k represents the different years (time):

$$O_{i,k}\sim \text{Poisson}(\mu_{i,k})$$ (1)

and

$$\mu_{i,k}=E_{i,k}\cdot \theta_{i,k}$$ (2)

where *E*_*i*,*k*_ is the expected cases in the i-th district and *θ*_*i*,*k*_ is the mean log relative risk (RR), which is as follows:

$$RR_{i,k}=e^{\theta_{i,k}}$$ (3)

which is modeled as follows:

$$log(\theta_{i,k})=\alpha +U_{i}+S_{i}+G_{k}+T_{k}+\delta_{i,k}$$ (4)

$$log\left(\theta_{i,k}\right)=\alpha +\beta_{j}\cdot X_{i,k,j}+U_{i}+S_{i}+G_{k}+T_{k}+\delta_{i,k}+\psi_{i,k}$$ (5)

Formula (4) is the spatiotemporal effect estimation model, where *α* is the intercept, *U*_*i*_ represents the spatially unstructured random effect, and *S*_*i*_ represents the spatially structured random effect. The prior distribution of the spatial structure effects was a conditional autoregressive process, considering the adjacent relationship (i.e., the incidence risk of HFMD in the adjacent area was more closely related, and the adjacency matrix W is an N × N-order matrix (N is the number of districts)). The value w_ij_ on the diagonal is 0. If district i and district j have a common boundary, w_ij_ is 1 and is 0 if they do not have a common boundary. *G*_*k*_ represents the temporal random effect, and $G_{k}\sim (0,\sigma_{k}^{2})$. *T*_*k*_ represents the time-structured random effect, which was assumed to follow a 1-order random walk. *δ*_*i*,*k*_ represents the spatiotemporal interaction term.

Formula (5) is the model to which the explanatory variable was added, where *X*_*i*,*k*,*j*_ is the value of the variable j in the k-th year of the i-th district; *β*_*j*_ is the regression coefficient corresponding to the variable j; *ψ*_*i*,*k*_ represents the random effect; and $\psi_{i,k,}\sim (0,\sigma_{i,k}^{2})$.

The explanatory variables selected in this study were mainly selected by reading the relevant literature [18–20,29,38] and combining that knowledge with the actual available data, including 11 variables such as the population, population density, GDP, per capita GDP, number of kindergartens, number of public kindergartens, number of private kindergartens, concentration of SO_2_, concentration of NO_2_, concentration of PM_10_, and concentration of O_3_.

We used the OpenBUGS software version 3.2.3 (MRC, UK, 2015) to develop a Poisson regression model under the region-level Bayesian framework to assess the spatial sociological effects of HFMD in Shenzhen city. The basic principle was to use Gibbs sampling to sample from the complete conditional probability distribution to generate the Markov chain. Then, through iteration, the model parameters were finally estimated. After running 1000 iterations of this experiment as the burn-in, a total of 15,000 iterations were run. To obtain a steady distribution, the convergence was evaluated by examining the trace plot of the samples for each chain. The deviance information criterion (DIC) values were used to compare the pros and cons of the different models and select the optimal model. The smaller the DIC value was, the better the goodness of fit of the model.

Finally, based on the determined optimal model, we used GeoBUGS software 1.2 to map the relative risk of HFMD for different districts in Shenzhen city. GeoBUGS is an add-on to OpenBUGS that fits spatial models and produces a range of maps as outputs.

## Results

### Descriptive statistics

**Fig 2:**
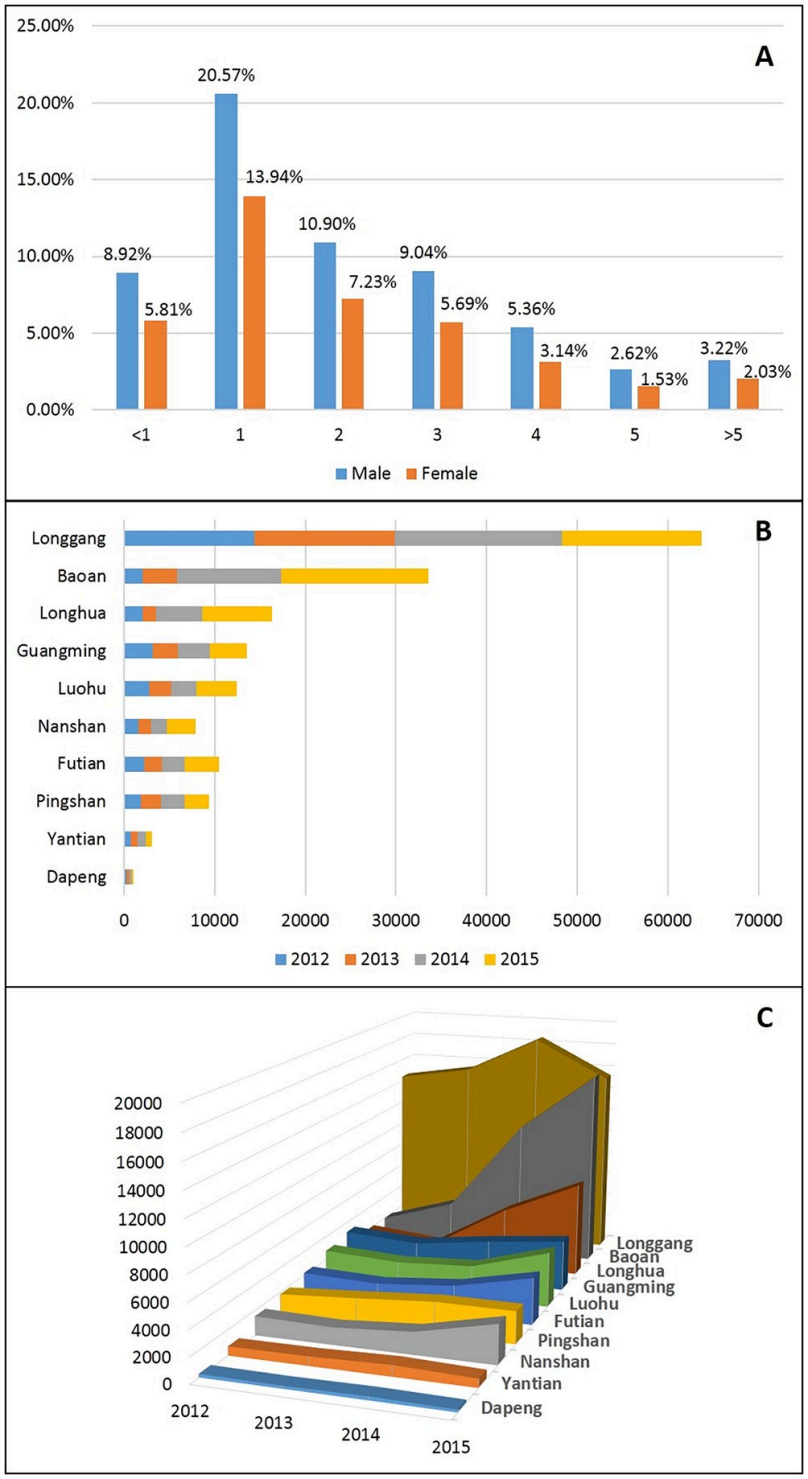
The statistical graph of the reported HFMD cases in Shenzhen, from 2012 to 2015. The characteristics distribution of age groups and sex, from 2012 to 2015(Fig.2.A). The distribution of the number of cases of each district from 2012 to 2015(Fig.2.B). The trends in the number of cases of each district from 2012 to 2015(Fig.2.C).

**Fig 3:**
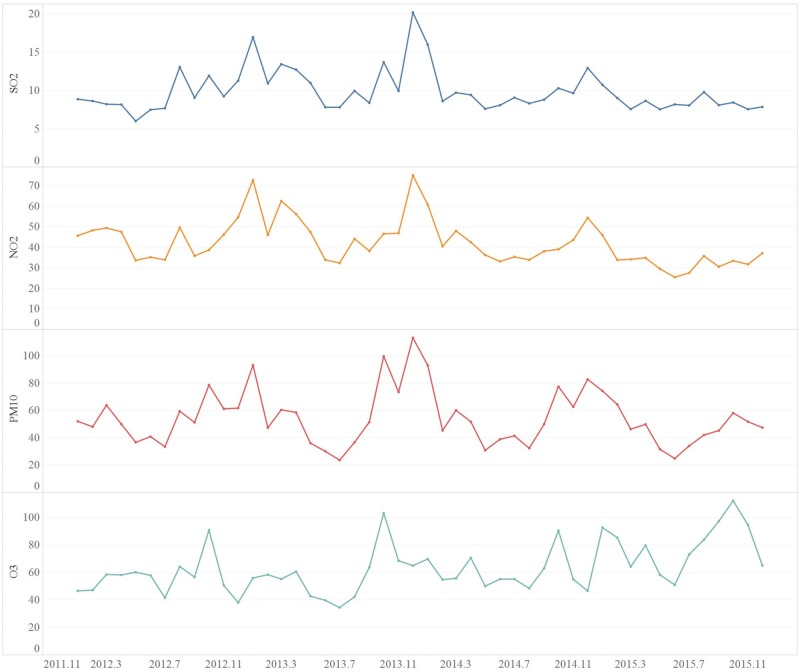
The variation of the pollutant over the 4 years, from 2012 to 2015.

A total of 171210 laboratory or clinically confirmed cases of HFMD in Shenzhen were reported from 2012 to 2015. There were 103802 male patients (60.63%) and 67408 female patients (39.37%). The main incidence group was the 1-year-old group (59096 cases; 34.52%), followed by the 2-year-old group (31042 cases; 18.13%). The median age was 2 years old, and the interquartile range was 1–3 years old. The specific age groups and sex distribution characteristics are shown in Table 1 and Fig 2A. The annual average incidences from 2012 to 2015 were 334.07/100000, 333.97/100000, 446.77/100000, 439.33/100000, respectively. A descriptive summary of the socioeconomic and air pollution variables is shown in Table 2. Fig 3 shows the variation in the pollutants over the 4-year period. In addition to the peak concentration of ozone in October, the peak concentrations of SO_2_, NO_2_ and PM_10_ were mainly during the winter from December to January. In the high incidence period of HFMD (May to July), they maintained a low concentration or showed a downward trend.

**Table 1:** Descriptive statistics of the basic characteristics of the HFMD cases [n (%)].

	<1-year-old	1-year-old	2-year-old	3-year-old	4-year-old	5-year-old	>5-year-old	Total
Male	15267(8.9)	35225(20.6)	18660(10.9)	15471(9.0)	9182(5.4)	4485(2.6)	5512(3.2)	103802(60.6)
Female	9948(5.8)	23817(13.9)	12382(7.2)	9735(5.7)	5378(3.1)	2625(1.5)	3469(2.0)	67408(39.4)
Total	25215(14.7)	59096(34.5)	31042(18.1)	25206(14.7)	14560(8.5)	7110(4.2)	8981(5.3)	171210(100)

*<1: A child who is less than 1 year of age; >5: A child who is greater than 5 years of age

**Table 2:** Descriptive statistics of the socioeconomic and air pollution variables.

Variables	Minimum	2.5% Percentile	Median	97.5% Percentile	Maximum
Population (100,000)(P)	1.31	3.23	10.42	14.39	28.63
Population Density (1000 person/ km^2^)(D)	0.44	2.87	5.63	8.15	18.31
GDP (¥10 billion)(G)	2.45	4.56	15.62	26.38	37.16
Per Capita GDP (¥10,000)(PCG)	7.52	11.13	15.16	20.39	30.50
Number of Kindergartens (per 10 kindergartens)(C)	1.10	4.00	14.45	18.33	35.80
Number of Public Kindergartens (C1)	1.00	2.00	4.00	9.00	18.00
Number of Private Kindergartens (per 10 kindergartens)(C2)	0.8	3.32	12.65	17.73	35.10
SO_2_ (mg/m^3^)	5.94	8.44	8.91	12.15	17.92
NO_2_ (mg/m^3^)	30.62	33.35	41.78	46.37	60.61
PM_10_ (mg/m^3^)	45.89	47.32	55.50	59.62	76.51
O_3_ (mg/m^3^)	40.48	54.69	61.37	78.91	79.58

### Spatiotemporal statistical analysis

This study selected data on the incidence of HFMD in 10 districts of Shenzhen from 2012 to 2015. The districts were Baoan, Nanshan, Longhua, Guangming, Longgang, Futian, Luohu, Yantian, Pingshan and Dapeng.

Through statistical analysis, a statistical graph of the annual number of cases was created (Fig 2). The overall number of cases of HFMD in Shenzhen showed a significant upward trend. As shown in Fig 2B, Longgang district had the largest number of cases, followed by Baoan district, and Dapeng district had the fewest cases. In addition, as shown in Fig 2C, the number of cases in Baoan district and Longhua district increased sharply from 2013–2015, while the number of cases in Longgang district peaked in 2014 but declined in 2015.

**Fig 4:**
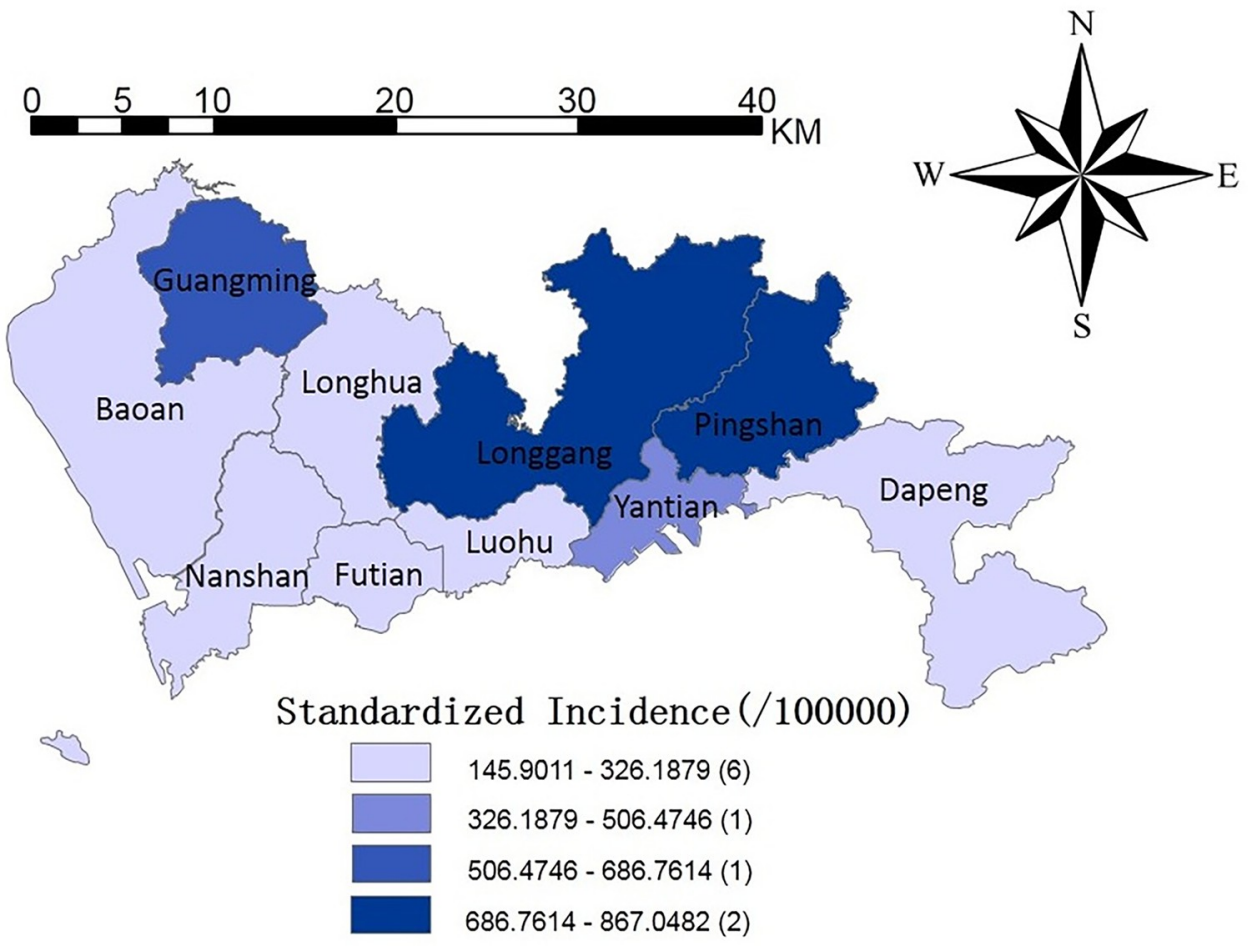
The Spatial distribution of standardized incidence rates of HFMD in each region in Shenzhen, from 2012 to 2015. This figure was generated using ArcGIS Geographic Information Systems software version 10.2 (ESRI, USA).

Due to the significant differences in the numbers of cases of HFMD and the populations in different areas, to spatially describe the areas with a higher risk of HFMD, we made a gradient diagram of the average incidence of HFMD in the various districts of Shenzhen (the number of cases of HFMD per 100000 people). Fig 4 shows the spatial distribution of the standardized incidence rates of HFMD in Shenzhen. The spatial distribution of the disease was mainly concentrated in northeastern Shenzhen city. According to the comparison of the standardized incidence rate of each region, the incidence rate of Longgang district was the highest, followed by Pingshan district and Guangming district.

### Spatiotemporal trend analysis

**Fig 5:**
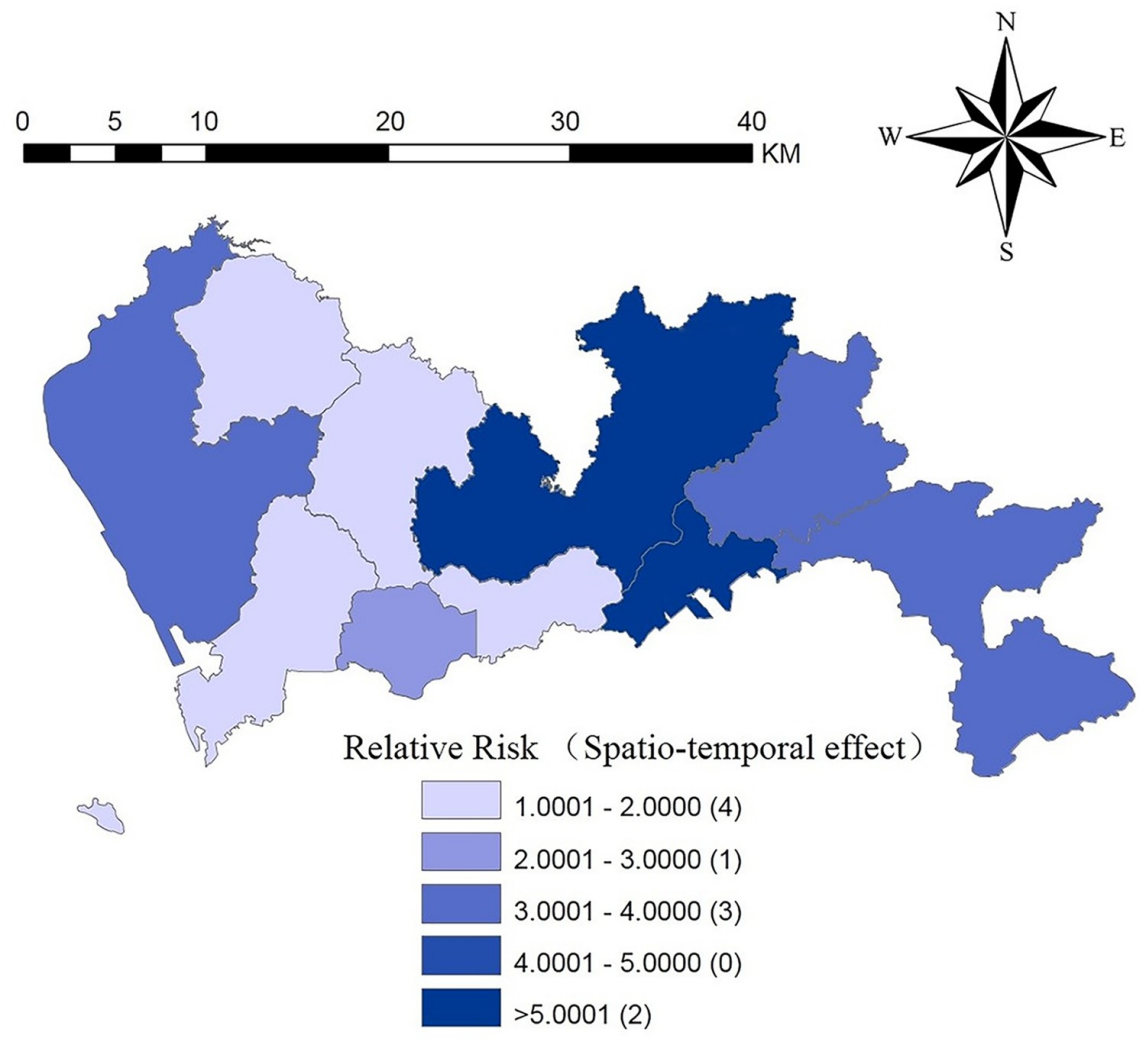
The spatial distribution of relative risk (only spatio-temporal effect) of HFMD in each region in Shenzhen. This figure was generated using ArcGIS Geographic Information Systems software version 10.2 (ESRI, USA).

We use the spatiotemporal effect estimation model (without explanatory variables) to estimate the risk of HFMD in various districts in Shenzhen and map the relative risk (RR), as shown in Fig 5. Compared with the direct use of the incidence of HFMD in each district, the Bayesian spatiotemporal model considered the proximity information, and its estimation results were more representative. It can be seen from Fig 5 that the relative risk (RR) value of Yantian district was the highest when only considering the spatiotemporal effects, and the areas with higher relative risk were mainly concentrated in the eastern part, while the risk in the western region was relatively low.

### Analysis of influencing factors

Due to the multicollinearity between the variables, multicollinearity analysis was used to calculate the variance inflation factor (VIF) between the 11 initial variables, and the variables with serious collinearity were excluded. It can be seen from Table 3 that the number of kindergartens (C) showed serious collinearity and were excluded from the model, so we cannot calculate its VIF value. Since the multicollinearity analysis only examined the integral correlation between the explanatory variables, the results were only used as a preliminary reference for the screening variables. The Bayesian model univariate regression results (Table 4) should be further selected as the criterion for selecting explanatory variables.

**Table 3:** Multicollinearity evaluation results (VIF: Variance inflation factor).

Variables	VIF	Variables	VIF
Population (100,000)(P)	11.45	Number of public kindergartens (C1)	5.87
Population Density (1000 Person/ km^2^)(D)	5.42	SO_2_ (mg/m^3^)	1.91
GDP (¥10 billion)(G)	1.58	NO_2_ (mg/m^3^)	4.89
Per capita GDP (¥10,000)(PCG)	2.04	PM_10_ (mg/m^3^)	7.57
Number of private kindergartens (per 10 kindergartens)(C2)	9.67	O_3_ (mg/m^3^)	2.35

*The number of kindergartens (C) showed serious collinearity and was excluded by the model.

**Table 4:** The regression coefficients of the univariate Bayesian models with respect to the different factor variables.

Variables	RR	95% *CI*
Population (100,000)(P)	0.98969	(0.98408,0.99835)
Population Density (1000 person/ km^2^)(D)	0.96027	(0.94960,0.97204)
GDP (¥10 billion)(G)	0.98040	(0.97638,0.98639)
Per capita GDP (¥10,000)(PCG)	0.97290	(0.96381,0.98340)
Number of Private Kindergartens (per 10 kindergartens)(C2)	1.01265	(1.00966,1.01922)
Number of Public Kindergartens (C1)	0.94763	(0.93995,0.96956)
SO_2_ (mg/m^3^)	0.95988	(0.94598,0.98719)
NO_2_ (mg/m^3^)	0.99264	(0.98764,0.99569)
PM_10_ (mg/m^3^)	0.99308	(0.98980,0.99644)
O_3_ (mg/m^3^)	0.99779	(0.99684,0.99956)

As seen from Table 4, during the period from 2012–2015, the factors related to the risk of HFMD included the population, population density, GDP, per capita GDP, number of private kindergartens, number of public kindergartens, concentration of SO_2_, concentration of NO_2_, concentration of PM_10_, and concentration of O_3_. We input the 10 variables that were meaningful according to the univariate Bayesian model analysis into the model for the next multivariate analysis. The DIC value of the model was 234.2. The smaller the value of DIC is, the better the goodness of fit of the model. The results of the multivariate analysis are shown in Table 5.

**Table 5:** Results of the regression coefficients for the multivariate Bayesian models with respect to the seven factor variables.

Variables	RR	95% CI
Population (100,000)(P)	1.00473	(1.00059,1.00761)
Population Density (1000 person/ km^2^)(D)	1.00010	(1.00002,1.00016)
GDP (¥10 billion)(G)	0.99897	(0.99662,1.00084)
Per Capita GDP (¥10,000)(PCG)	0.98840	(0.98660,0.99026)
Number of Private Kindergartens (per 10 kindergartens)(C2)	0.99998	(0.99995,1.00003)
Number of Public Kindergartens (C1)	0.97686	(0.96946,0.98403)
SO_2_ (mg/m^3^)	1.00215	(1.00170,1.00232)
NO_2_ (mg/m^3^)	1.00058	(1.00028,1.00078)
PM_10_ (mg/m^3^)	0.99108	(0.98551,0.99840)
O_3_ (mg/m^3^)	0.99587	(0.99534,0.99619)

From 2012–2015, the relative risk factors of HFMD were as follows: population, population density, per capita GDP, number of public kindergartens, concentration of SO_2_, concentration of NO_2_, concentration of PM_10_, and concentration of O_3_. GDP and the number of private kindergartens had no significant effect on the risk of HFMD. The population, population density, concentration of SO_2_ and concentration of NO_2_ were positively correlated with the risk of HFMD. The relative risks were 1.00473 (95% *CI*: 1.00059–1.00761), 1.00010 (95% *CI*: 1.00002–1.00016), 1.00215 (95% *CI*: 1.00170–1.00232) and 1.00058 (95% *CI*: 1.00028–1.00078), respectively. The per capita GDP, number of public kindergartens, concentration of PM_10_ and concentration of O_3_ were negatively correlated with the risk of HFMD. The relative risks were 0.98840 (95% *CI*: 0.98660–0.99026), 0.97686 (95% *CI*: 0.96946–0.98403), 0.99108 (95% *CI*: 0.98551–0.99840) and 0.99587 (95% *CI*: 0.99534–0.99610), respectively.

**Fig 6:**
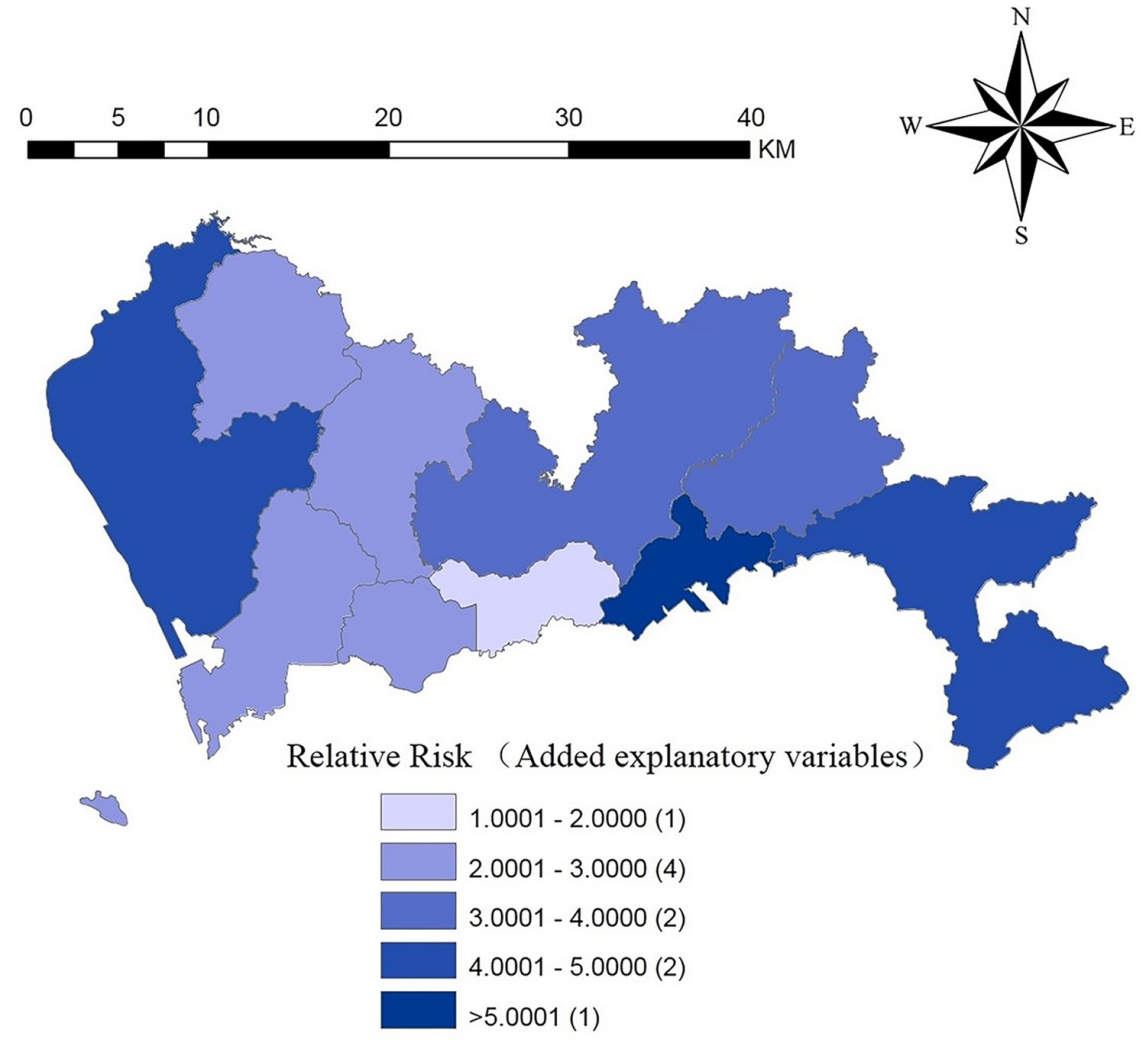
The Spatial distribution of relative risk (added explanatory variables) of HFMD in each region in Shenzhen. This figure was generated using ArcGIS Geographic Information Systems software version 10.2 (ESRI, USA).

Finally, we calculated the relative risk of HFMD in the various regions of Shenzhen and plotted the relative risk map, which is shown in Fig 6. Yantian district had the highest relative risk, which meant that the risk of incidence in Yantian district was the highest, followed by Baoan district.

The variation between Figs 4 and 6 shows that the model was gradually optimized or the prediction accuracy was improved. In Fig 4, we hope to judge the future incidence trends by comparing the average incidences of each district of Shenzhen from 2012–2015. However, such a prediction was not sufficiently accurate. Therefore, we considered the effects of the spatiotemporal interactions of the disease (Fig 5). Fig 5 showed slight changes in the tendency of the disease and high-risk areas compared to Fig 4. In Fig 6, we not only retained the spatiotemporal interaction effect but also added the effects of the influencing factors.

To demonstrate the validity of the model, we obtained the RR values (RR_ik_) for each area of each year through the model. Then, we made the point diagram of the RR values (RR_ik_) of each region over the four years (2012–2015) to reflect the change during the time scale. On this basis, the fitting trend line of each region was increased (the goodness of fit R^2^ was more than 0.8), and the RR values of each region in 2016 were estimated. Finally, the ranking of the RR values were compared with the ranking of the incidence of each region in 2016.

In 2016, the top five high-incidence areas in Shenzhen were Guangming, Baoan, Longhua, Pingshan and Longgang, which had incidence rates of 492.70/100000, 426.12/100000, 415.88/100000, 367.31/100000, 323.28/1000000, respectively. The top five high-RR values areas were Yantian, Baoan, Guangming, Pingshan and Longgang, which had values of 5.28, 4.62, 3.59, 3.76 and 3.27, respectively. Although there were minor differences in the individual values, the basic order of the first five was consistent.

## Discussion

As a highly developed first-tier city in China, Shenzhen is also a hub linking mainland China and Hong Kong. The city belongs to the subtropical climate region, and it is highly populated, highly mobile, and crowded from housing and traffic and has a shrinking living space. These characteristics cause Shenzhen to have a high frequency of infectious diseases, such as HFMD [40–43]. This study included 171,210 cases of HFMD in Shenzhen from 2012 to 2015. The Bayesian spatiotemporal model was used to analyze the factors that influenced the incidence of HFMD in Shenzhen, including the population factors (population and population density), economic factors (GDP and GDP per capita), educational resource factors (public, private and total numbers of kindergartens), and environmental pollution factors (the concentrations of SO_2_, NO_2_, PM_10_, and O_3_). By testing the optimal model, the relative risk map of each district was drawn to predict the high-risk areas of HFMD and the future trend of the disease in Shenzhen.

The results of the study indicated that the population and the population density were positively correlated with the risk of HFMD. HFMD is an infectious disease, and an increase in population size or population density may increase the risk of disease transmission, which is consistent with national research results [29]. The per capita GDP was negatively correlated with the risk of HFMD. This may be because Shenzhen’s overall economic level is good. In general, the children living in areas with social and economic benefits were less likely to be infected with HFMD due to the availability of local health care and better sanitation [29,38].

In China, children in kindergarten are usually aged 3–6, and this age group is most vulnerable to HFMD. Although the number of private kindergartens was not related to the incidence of HFMD, the number of public kindergartens was negatively correlated with the incidence of HFMD. At present, as far as Guangdong Province is concerned, public kindergartens generally have stronger financial support and more standardized management systems. Therefore, in general, the sanitary environment of public kindergartens is better, leading to a decreased risk of disease transmission [44,45].

The study found that SO_2_ increased the risk of HFMD, with an RR of 1.00215 (95% *CI*: 1.00170–1.00232). However, we still do not know the exact mechanism by which exposure to SO_2_ increases the risk of HFMD in children. However, from a biological point of view, HFMD can be transmitted through the respiratory tract, and SO_2_ also has a strong stimulating effect on the respiratory mucosa, which can induce oxidative stress and systemic inflammation or increase the permeability of the solute [18,46]. This increases the susceptibility of children to HFMD. The study also found that NO_2_ increased the risk of HFMD, which was consistent with the results of another Shenzhen air pollution study [47]. Although no laboratory studies were found on the direct effect of NO_2_ on enterovirus, it has been confirmed that the NO_2_ concentration was positively correlated with the incidence of rotavirus [48]. Another study also confirmed that NO_2_ may have direct effects on epithelial cells, cause systemic inflammation and immune activation and modulate the intestinal microbiota [49].

The study also found that high concentrations of ozone play a protective role. At present, research on the relationship between ozone concentration and infectious diseases is still limited. Although some of the basic mechanisms of action of ozone in pulmonary toxicology and in medicine have been clarified, and the absolute view of ozone toxicity (that ozone is toxic and harmful to humans) is incorrect [50–53]. To judge its toxicity, it is necessary to consider the exposure dose, molecular structure and the susceptibility of individuals to exposure at the same time. At present, some studies have been based either on lungs or on studies performed in artificial environments that do not correspond to the real antioxidant capacity of the body compartments [50]. Ozone doses that are well calibrated against the potent antioxidant capacity of blood can trigger several useful biochemical mechanisms and reactivate the antioxidant system [51]. Ozone may inhibit the ability of viruses to survive or replicate in the external environment, affecting the spread of HFMD [54]. Studies have also confirmed that appropriate ozone concentrations limit the production of viruses, prolong the survival time of cells, and inhibit the production of the cytokines related to EV 71 infection [55].

In addition, the study found that the concentration of PM_10_ was inversely related to the risk of HFMD, and this result was consistent with the research results of the relationship between PM_10_ and HFMD in Yuexiu district from 2010 to 2011 [56]. At present, the exact mechanism of the relationship between exposure to PM_10_ and the risk of HFMD in children is still unknown. However, studies in Nanjing, Beijing, and Guangzhou have shown that public perception and acceptable risk levels of air pollution can prompt individual behavioral changes (risk reduction behaviors) [57–61]. When the haze weather is severe or the AQI (air quality index) is large, the public might reduce the number of outings, wear masks when going out, use air purifiers, reduce the number of open windows, and increase the frequency of cleaning mouths and noses. These behaviors reduce exposure to environmental pollution, thus reducing the risk of HFMD [57,62]. More studies have confirmed that families with children, in particular, are more likely to engage in these risk reduction behaviors [57,60]. In addition, the negative correlation with PM_10_ may also be related to the special geographical environment of Shenzhen. Shenzhen is located between 113°46’-114°37’ east longitude and 22°27’-22° north latitude. It is close to the sea, has a warm climate, and is humid and rainy. Warm and humid sea breezes are very beneficial to the diffusion and deposition of pollutants [63].

This study is the first a Bayesian spatiotemporal model has been used to analyze the impact of socioeconomic factors and air pollution factors on the risk of HFMD from urban spatial dimensions. Second, this study established a more mature Bayesian spatiotemporal prediction model for HFMD that can calculate the risk of HFMD in each area, to identify high-risk areas. However, these high-risk areas are not fixed, and as long as the original data are updated, the relative risk of each district will be updated, resulting in changes in the high-risk areas. However, for the predicted high-risk areas, we can implement preventive measures in a timely manner to reduce the harm of the disease. Therefore, early discovery, early prevention and effective protection of children’s health are needed.

The risk of disease in Yantian district was the largest when only the spatiotemporal effects were analyzed. In Fig 4, from the spatial scale, we can see that the high-risk areas from 2012 to 2015 were mainly concentrated in northern Shenzhen (Longgang district, Pingshan district). The risks of disease in Longgang district and Pingshan district decreased, while the risk of disease in Baoan district increased.

There were some limitations to our research. First, some patients have mild symptoms and do not go to the hospital for treatment. Our reporting system does not record such self-treating individuals, so the incidence may be underestimated. The use of clinical diagnosis rather than laboratory diagnosis in most cases is another source of reporting bias. Second, only 4 years of data were analyzed in this study, and there was a certain degree of collinearity between the variables, both of which may reduce the stability of the model. Third, the purpose of this study was to explore the relationship among the socioeconomic factors, the air pollution factors and the incidence of HFMD, which cannot be used as evidence to judge a causal relationship. Further studies are needed to explore the physiological mechanisms of the effects of air pollution on HFMD and whether other air pollutants may affect the incidence of HFMD in children.

### Conclusion

Studies have confirmed that socioeconomic factors and air pollution factors have an impact on the incidence of HFMD in Shenzhen, China. Among these factors, the population, the population density, the concentration of SO_2_ and the concentration of NO_2_ were positively correlated with the risk of HFMD, and the per capita GDP, the number of public kindergartens, the concentration of PM_10_ and the concentration of O_3_ were negatively correlated with the risk of HFMD. According to the map of relative risk obtained from the Bayesian spatiotemporal multifactor model, the risk of Longgang district and Pingshan district decreased, while the risk of Baoan district increased. The results will have important practical implications for local authorities, which is conducive to accurate prevention and can be used to formulate targeted disease intervention measures.
